# Early diagnosis of Alzheimer's disease based on brain morphological changes: A comprehensive approach combining voxel-based morphometry and deep learning

**DOI:** 10.1016/j.ynirp.2025.100315

**Published:** 2026-01-06

**Authors:** Mohammad Rezaei, Shaghayegh Mohammadikhaveh, Hadis Faraji, Ramin Ardalani, Mina Rezaei, Alireza Shirazinodeh

**Affiliations:** a Department of Biomedical Engineering, Tehran Azad University of Medical Sciences, Tehran, Iran; b School of Engineering Science, College of Engineering, University of Tehran, Tehran, Iran; c Department of Biomedical Engineering and Medical Physics, School of Medicine, Shahid Beheshti University of Medical Sciences, Tehran, Iran; d Department of Mathematical and Statistical Sciences, Southern Illinois University, Carbondale, USA

**Keywords:** Voxel-based morphometry, Early and late MCI, T1-weighted MRI scans, Alzheimer's Disease, Convolutional neural networks, Fully connected neural network

## Abstract

Deep learning algorithms optimize data by enhancing resolution and suppressing noise associated with biological knowledge. The root issue is that, for example, CNNs learning mathematical patterns from statistical correlations in the data without regard to biological cues whatsoever, and merely apply filters such as max pooling, never grasping what the biological cues they are supposed to investigate are. This blind procedure can indeed be in technical language; however, it does not help to identify meaningful insights into neuroimaging, where interpretability is essential, and such inadequacies pose a grave challenge. In our research, rather than depending on the CNNs and FCNs only for the feature extractions, we have integrated biologically motivated features into voxel-based morphometry as well as deep learning. Our goal is to analyze T1-weighted MRI scans and T2-Flair images to investigate the characteristics of gray matter, white matter, cerebrospinal fluid, and white matter Hyperintensity in patients with mild cognitive impairment (MCI) who lie on the spectrum between normal aging and Alzheimer's disease (AD). So we extracted critical structural features such as white matter Hyperintensity, gray matter volume, white matter volume, cerebrospinal fluid (CSF) volume, and cortical thickness. These are biologically meaningful biomarkers that reflect the neurodegenerative alterations directly. To validate our method, after the detection of biological features, we have converted them into 3-bit, 4-bit, 8-bit, and 16-bit images. These images were used as inputs for both FCN and CNN models to investigate the early symptoms of AD from classified intracranial features.

## Highlights

• We have used the voxel-based morphometry images as inputs to deep learning algorithms that are mentioned in the manuscript, to identify meaningful insights into the neuroimaging procedure in the early detection of AD.
• Our research uses T1-weighted MRI scans from the Alzheimer's Disease Neuroimaging Initiative (ADNI), with structural images from subjects across five clinical groups (CN, EMCI, MCI, LMCI, AD). We derived five well-established imaging measures: total White Matter Hyperintensity (WMH), gray matter (GM) volume, white matter (WM) volume, cerebrospinal fluid (CSF) volume, and cortical thickness, from SPM12 and the CAT12 toolbox.
• These measures were transformed into multi-bit (3, 4, 8, and 16-bit) heatmap representations, providing dense, visually encoded inputs for convolutional and fully connected neural networks. Our results indicate that these biologically driven heatmaps provide excellent classification performance, illustrating the complementary value of multimodal structural biomarkers.

e. For the Alzheimer's Disease Neuroimaging Initiative^1^

## 1 Introduction

Alzheimer's Disease (AD) is widely acknowledged as the primary cause of dementia worldwide (Self et al., 2023). The population affected by AD is expected to grow significantly, reaching about 78 million in 2030 and potentially expanding to 139 million by 2050 (Reitz et al., 2023). A key pathological hallmark of AD is the extensive shrinkage of the brain, with the medial temporal lobe structures being among the first and most affected areas (Braak et al., 1999; Fox et al., 1996). AD involves changes in both Gray Matter (GM) and White Matter (WM), and these abnormalities have been associated with cognitive decline (Rose et al., 2000; Bozzali et al., 2001, 2002). Neuroimaging techniques have been extensively used to monitor brain shrinkage and follow the initiation and advancement of neurodegenerative disorders such as AD (Callen et al., 2001; De Santi et al., 2001; Du et al., 2001; Soininen et al., 1994). Long-term studies have been instrumental in identifying brain volume changes during typical aging (Resnick et al., 2003) and tracking AD-related neuropathology's progression over time (Driscoll et al., 2009; Fox et al., 2000; Clifford et al., 2004; Misra et al., 2009; Mungas et al., 2005; Schott et al., 2005). GM and WM volumes tend to decrease significantly over time in older people, even those who are extraordinarily healthy, as part of the typical aging process (Resnick et al., 2003). Research has shown that assessing cortical thickness in specific brain areas could be an effective tool for identifying structural changes in cognitively normal individuals at risk for AD (Burggren et al., 2008) and in those with Mild Cognitive Impairment (MCI) (Singh et al., 2006). Moreover, a decrease in cortical thickness appears to closely relate to the severity of clinical symptoms, even in the early stages of AD (Dickerson et al., 2009). This suggests that cortical thickness might be a more sensitive and complementary measure of early pathological changes than standard MRI-based volumetric analysis for individuals at risk of cognitive decline. However, these studies are cross-sectional and, therefore, cannot fully address the long-term effects of age-related changes in cortical thickness. Structural MRI captures both processes: cortical thickness, “AD-signature” atrophy indexes neurodegeneration, White Matter Hyperintensities (WMH) index small-vessel disease, and ventricular Cerebrospinal Fluid (vCSF) expansion reflects global tissue loss. Longitudinal evidence indicates that vCSF growth sensitively tracks clinical progression and links to cognition; notably, periventricular WMH relate more strongly to subsequent ventricular expansion than deep WMH, tying small-vessel injury to accelerating atrophy (Adamo et al., 2020). A robust methodological literature shows that WMH can bias gray matter measurements on T1-weighted MRI. Because WMH appears hypo-intense on T1, it can misclassify WMH as gray matter, inflating cortical volumes and distorting structure effects that are especially pronounced in the caudate (Dadar et al., 2021). Classic work quantified the magnitude of this problem: in patients with severe WMH, approximately 4–5 % of brain voxels were misclassified, gray matter was overestimated by 6 %, white matter was underestimated by 7–8 %, and lobar gray matter inflation (frontal temporal) was common (Levy-Cooperman et al., 2008). Taken together, the evidence motivates an approach that models longitudinal MRI to capture the shifting salience of biomarkers, from early cortical thinning to later ventricular expansion, while explicitly accounting for WMH both as a biologically meaningful signal and as a potential source of T1-based measurement bias. In our proposed method and in the level of preprocessing, based on the recent research (Dadar et al., 2021; Levy-Cooperman et al., 2008; Adamo et al., 2020), we therefore focus on cortical thinning, WMH burden, and vCSF expansion as complementary markers spanning neurodegeneration and small-vessel disease, and we adopt analysis choices that are robust to WMH-related misclassification.

One of the focuses of attention among these is the identification of biomarkers for early Alzheimer's disease from MRI scans (Ebrahimighahnavieh et al., 2020). In analyzing complex visual data, Deep Neural Networks (DNNs) have shown that they are superior and faster in the early stages of diagnosis than conventional image processing techniques (Ahn and Kwak, 2018). In particular, Convolutional Neural Networks (CNNs) allow the potential to distinguish important features in Regions of Interest (ROI) such as GM-atrophy and cortical thinning, enabling an accurate representation of class-specific patterns (Marwa et al., 2023). Janghel and Rathore have noted in their study that the VGG-16 deep learning architecture obtained an average classification accuracy of 99.95 % in the Alzheimer's disease data set, which could also classify Alzheimer's Disease and Control Normal (CN) individuals (Janghel and Rathore, 2021). Furthermore, according to the research by Basaia et al., the CNN method achieves an acceptable accuracy, sensitivity, and specificity of above 98 % in the classification of Alzheimer's disease in the ADNI and Milan datasets. Also, CNN showed over 86 % accuracy in the diagnosis of c-MCI from the elderly controls and 75 % accuracy in c-MCI from s-MCI (Basaia et al., 2019). In this way, Fully Convolutional Networks (FCNs) augment the CNN method by adding fully dense pixel-wise image segmentation capabilities. This development opens the possibility of accurate measurements, which is important in the correct diagnosis of Alzheimer's disease. Deng and Wang have confirmed these findings, showing how FCNs may significantly enhance the performance of various types of diagnosis of Alzheimer's disease (Deng et al., 2023).

In this work, we present an integrated framework employing the SPM-CAT12 and LST toolboxes on T1-weighted MRI scans and T2-Flair, based on voxel-based morphometry and deep learning algorithms. The structural features include Total Intracranial Volume (TIV), gray matter volume, white matter volume, cerebrospinal fluid volume, cortical thickness, and White-matter Hyperintensity, which were extracted and analyzed across five cognitive health groups: CN, Early-MCI (EMCI), MCI, Late-MCI (LMCI), and AD. These features are then transformed into image representations to take advantage of the CNNs and FCNs.

## 2 Materials and methods

Early diagnosis and classification of Alzheimer's disease can have a significant impact on effective intervention. Artificial intelligence plays a crucial role in enhancing performance and improving classification accuracy, but this often depends on how the input images are preprocessed.

### 2.1 Participants and imaging data

**Fig. 1:**
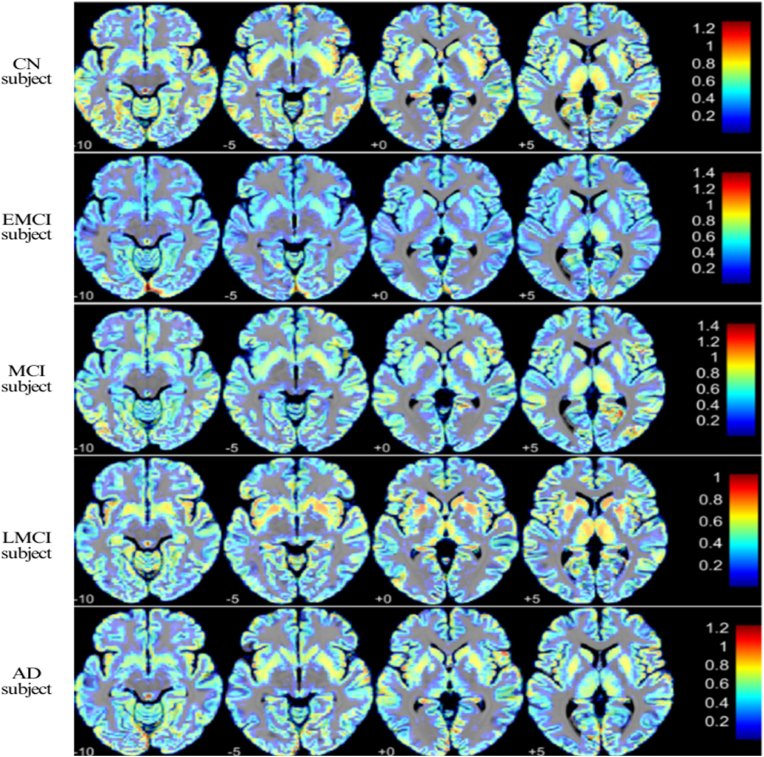
Comparison between the brain structures of CN, EMCI, LMCI, and AD subjects.

The analytic dataset comprised 114 unique participants contributing 625 T1-weighted sessions across five diagnostic strata: CN (22 participants, 113 scans), EMCI (20 participants, 101 scans), LMCI (25 participants, 188 scans), MCI (25 participants, 147 scans), and AD (22 participants, 76 scans). Analyses focused on ADNI phases 2 and 3 to ensure sufficient longitudinal follow-up and harmonized multi-site acquisition for the questions addressed here. Longitudinal coverage (sessions per participant) and follow-up durations are summarized in Table 1. ADNI acquisitions follow harmonized multi-site 3D T1-weighted MP-RAGE–type protocols; across phases, imaging was performed on 1.5 T and 3 T systems (ADNI-3 exclusively 3 T), using standardized sequence parameters and site calibration procedures. We analyzed de-identified MRI data from the Alzheimer's Disease Neuroimaging Initiative (ADNI; public–private partnership led by Michael W. Weiner, MD). ADNI was established to determine whether serial MRI, PET, fluid biomarkers, and clinical/neuropsychological assessments can be combined to measure disease progression from MCI to Alzheimer's disease. All ADNI participants provided written informed consent under protocols approved by each site's IRB. The Comparison between the brain structures of CN, EMCI, MCI, LMCI, and AD subjects is shown in Fig. 1.

**Table 1:** Demographic and clinical characteristics of the analytic sample across diagnostic groups. Values represent mean ± standard deviation unless otherwise indicated. MMSE = Mini–Mental State Examination; CDR-SB = Clinical Dementia Rating – Sum of Boxes. Session counts and follow-up reflect the longitudinal coverage of each cohort.

Group	Participants (n)	Age (years), mean ± SD	Education (years), mean ± SD	MMSE (baseline), mean ± SD	CDR-SB (baseline) mean ± SD	Scans (n)	Sessions median	Follow-up (months), median [range]
CN	22	71.3 ± 5.3	17.1 ± 2.2	29.23 ± 0.92	0.00 ± 0.00	113	4 [4–5]	78.7 [25.3–138.7]
EMCI	20	69.0 ± 6.2	16.6 ± 3.0	28.45 ± 1.64	1.10 ± 0.85	101	4 [3–7]	84.2 [3.0–141.7]
LMCI	25	68.0 ± 9.1	16.0 ± 2.5	27.52 ± 1.83	1.60 ± 1.09	188	7 [6–9]	72.6 [12.2–132.9]
MCI	25	72.3 ± 6.1	16.8 ± 2.7	28.58 ± 1.56	1.35 ± 1.19	147	5 [3–9]	67.3 [11.3–171.0]
AD	22	74.5 ± 6.3	15.3 ± 2.5	23.18 ± 2.79	4.43 ± 1.51	76	3 [3–4]	25.2 [12.3–95.8]

### 2.2 Data processing

All structural MRI underwent a two-stage pipeline designed for aging cohorts in whom WMH are common and can distort tissue labeling. In the first stage, we rigidly coregistered each session's T2-FLAIR to its native T1, then segmented WMH with an SPM-based approach, and designed the corresponding masks to perform lesion filling on the T1-weighted images before any morphometry. This WMH-aware correction reduces misclassification at the gray and white boundary and stabilizes subsequent thickness and volumetric estimates. In the second stage, we performed all downstream morphometric processing in SPM12/CAT12 on the lesion-filled T1 volumes using a fully scripted workflow.

**Fig. 2:**
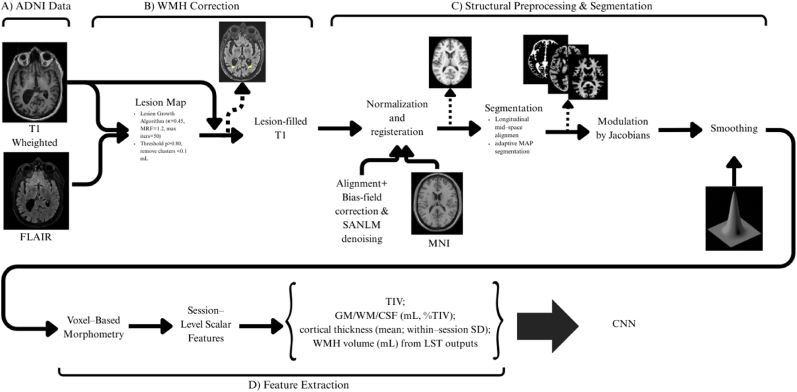
End-to-end structural MRI pipeline. a. Original Images from ADNI Database: T1-weighted and T2-FLAIR Scans. b. WMH correction stage, T2-FLAIR is coregistered to T1, white-matter hyperintensities are segmented using LST (Lesion Segmentation; κ = 0.45, MRF = 1.2, max iterations = 50), maps are thresholded (p > 0.80) with clusters smaller than 0.1 mL removed. c. Structural preprocessing and segmentation: subject alignment to a mid-space, bias-field correction, SANLM denoising, nonlinear normalization to MNI space, adaptive MAP tissue segmentation, modulation by Jacobians, and smoothing. d. Session-level features: TIV; GM/WM/CSF absolute volumes and percentage of TIV; global cortical thickness (session mean and within-session SD); and WMH volume from LST outputs. e. Features (and modulated maps for VBM) are then provided to the CNN stage for downstream modeling.

(i) within-subject rigid alignment to a mid-space for longitudinal consistency,
(ii) bias-field correction and spatially adaptive denoising to improve tissue contrast,
(iii) probabilistic tissue segmentation with partial-volume modeling and total intracranial volume estimation,
(IV) and nonlinear normalization to MNI space with preservation of local volume via Jacobian modulation and conventional smoothing for voxel-wise inference. The processing workflow is summarized in Fig. 2.

#### 2.2.1 WMH segmentation and lesion filling

Because ADNI participants are older and often carry some white-matter hyperintensities (WMH), we first corrected T1-weighted scans for WMH-related misclassification. After registration of the T2-FLAIR images to their native T1, we segmented WMH with the Lesion Segmentation Toolbox (LST; “Lesion Growth Algorithm”, LGA) using the following parameters: initial threshold κ = 0.45, MRF = 1.2, and maximum iterations = 50. To minimize false positives in controls, lesion probability maps were thresholded at p > 0.8, and clusters smaller than ∼0.1 mL were discarded. Before filling, to avoid edge under-filling, we lightly dilated (1 voxel) lesion masks. The resulting lesion masks were used to perform lesion filling on the native T1 image (LST's Lesion Filling module), replacing lesion voxels with intensities estimated from surrounding normal-appearing white matter. All downstream morphometric steps were run on these lesion-filled T1 volumes. We also retained total WMH volume per session for sensitivity analyses.

#### 2.2.2 Structural preprocessing and segmentation

We carried out morphometric processing in SPM12 on the lesion-filled T1-weighted volumes. For participants with repeated sessions and to avoid bias toward any single scan and to maximize sensitivity to subtle longitudinal change, we first rigidly aligned images from all time points within the subject and brought them to a subject-specific mid-space. We then corrected residual intensity non-uniformity and applied a spatially adaptive non-local means (SANLM) denoising filter to suppress high-frequency noise while preserving tissue boundaries. Tissue classification proceeded with a probabilistic adaptive.

MAP framework that models partial-volume effects; gray matter, white matter, and CSF probability maps were produced and morphologically cleaned, and total intracranial volume (TIV) was estimated for use as a covariate. Finally, we normalized the resulting tissue maps to MNI space with a diffeomorphic approach (shooting/DARTEL family); therefore, the corresponding deformation fields were retained. Spatial resampling used 1.5-mm isotropic voxels with 4th-degree B-spline interpolation; individual flow fields were retained to enable ROI warping and surface summaries. Modulation by Jacobians preserved local volume before smoothing. For voxel-based morphometry, we modulated the maps by the Jacobian determinants of these deformations to preserve local volume, resampled to 1.5-mm isotropic resolution, and then we smoothed them with an 8-mm FWHM Gaussian kernel to accommodate inter-subject anatomical variability and improve SNR. Throughout the pipeline, we performed visual quality control of alignment, segmentations, and normalized outputs, and reviewed objective homogeneity indices; scans flagged as outliers were re-checked and, when necessary, reprocessed.

### 2.3 Feature extraction and group comparisons

From each lesion-filled T1-weighted session, we derived a compact set of structural features intended to capture global tissue composition, cortical integrity, and the burden of presumed small-vessel disease. Potentially, probabilistic tissue maps provide absolute gray-matter, white-matter, and cerebrospinal fluid volumes (mL) and their relative fractions with respect to TIV (%TIV), so we estimated TIV for covariate control. Next, we computed a global cortical thickness summary from surface-based estimates (session-level mean thickness). From the lesion-segmentation outputs, we obtained the WMH volume (mL) and treated it as a complementary descriptor of small-vessel disease load that may influence tissue contrasts and thickness estimates.

In the next process of feature extraction and in the stage of voxel-based morphometry (VBM), we modulated, spatially normalized GM maps (1.5-mm isotropic resolution; 8-mm FWHM smoothing) and carried forward to voxel-wise inference. The scalar features (TIV, GM/WM/CSF absolute and relative volumes, global cortical thickness, and WMH volume) supported cohort description and served as candidate predictors in subsequent learning analyses. Group-wise descriptive statistics (numbers of participants and scans, follow-up time, and central tendency/dispersion for TIV, GM/WM/CSF, global thickness, and WMH) are reported in Table 2. Moreover, diagnostic categories followed ADNI conventions, including the EMCI vs. LMCI distinction to index milder episodic-memory impairment at enrollment. These tiers differ in biomarker positivity and clinical trajectory and were therefore analyzed separately.

**Table 2:** Extracted features from MRI data and their associated applications.

Feature	Description	Application
TIV	Computed to serve as a covariate in analyses, ensuring that volume differences are not due to variations in head size	TIV computation is used to control for head size differences in statistical analysis.

WM Volume	Absolute and relative GM volumes are quantified	Included Hyperintensity mapping

GM Volume	Absolute and relative GM volumes are measured	GM maps segmentation

CSF Volume	Measured CSF volume, normalized relative to TIV.	Analyzed changes to infer brain atrophy, as increased CSF volume is associated with GM and WM loss

Cortical Thickness	The estimated thickness of the cerebral cortex in a GM voxel, projected onto the cortical surface.	Divide the MCI group into two parts based on the cortical thickness.

For inferential comparisons on scalar features, we used general linear models with Group as the factor of interest and the following covariates, unless otherwise noted: age, sex, and TIV (the latter omitted when modeling relative volumes or thickness). Given the multi-site nature of ADNI, scanner/site was included as a nuisance term when available. WMH volumes showed a positive skew; accordingly, we applied a log(1+WMH) transform for hypothesis testing and reported the back-transformed group means for interpretability. Pairwise contrasts were adjusted for multiple comparisons using the false discovery rate (FDR) across feature families.

For VBM, we implemented a voxel-wise GLM with the same covariates, assessed effects with random-field theory–based family-wise error control at p < 0.05 (cluster-forming threshold p < 0.001, and summarized peak/cluster extents alongside region-of-interest averages for interpretability. Longitudinal data were analyzed at the session level; to respect repeated measurements, standard errors were clustered by subject (equivalently, a subject-level random intercept yields the same inference for fixed effects in this setting). Sensitivity analyses using non-parametric rank-based tests (for scalars) and permutation-based threshold-free cluster enhancement (for VBM) confirmed the robustness of the principal results.

### 2.4 Statistical analyses

We have undertaken Statistical analyses to characterize cross-sectional differences in five structural measures: CSF, GM, WM, cortical thickness (CT), and white-matter Hyperintensity volume (WMH), across the diagnostic groups (CN, EMCI, MCI, LMCI, AD). For transparency and context, descriptive statistics (mean and standard deviation) are summarized for each variable in each group before inferential testing. Because parametric between-group comparisons assume distributional normality, we first evaluated the normality of each variable within each group using the Shapiro–Wilk test. The large majority of variable–group combinations violated normality at α = 0.05, with only a few instances in the AD cohort approaching approximate normality. Given these diagnostics, we proceeded with nonparametric omnibus testing using the Kruskal–Wallis test to compare the five groups on each measure, reporting χ^2^ statistics with associated p-values.

### 2.5 Converting features into multi-bit heatmaps

We have introduced an innovative approach by transforming numerical clinical features into image-based representations (heatmaps) rather than using raw numerical data. First, we have extracted five quantitative features: CSF, CT, GM, WM, and WMH. To account for head-size variability, all features are expressed relative to total intracranial volume (TIV). In addition, we have defined a temporal variable called ΔT_months. For each subject, the first scan was set as baseline (0), and each subsequent scan was given a value equal to the number of months from that baseline. This approach allowed us to consistently track the temporal progression of each subject's data. To organize the data further, we have used a hierarchical labeling system in the format Group Label. Subject Number. ScanNumber (e.g., 0.1.1 for the first scan of the first AD subject). Group labels were defined as AD = 0, CN = 1, MCI = 2, EMCI = 3, and LMCI = 4. Finally, all processed data were combined into a single dataset for analysis. This hierarchical structure can track changes both within individuals and between groups and overall trends. This lets us observe how each subject changes over time (for example, comparing scans 0.1.1 with 0.1.2 for subject 0.1), and compare subjects or groups (like Alzheimer's disease vs. healthy controls). This fine detail is essential for long-term studies of disease progression or treatment effects. As mentioned earlier, we defined a heatmap for each network input. The x-axis represents time; we use 3-month bins from 0 to 84 months (28 bins in total), based on the mean time points indicated in the preprocessing stage. The y-axis represents the normalized features. Each heatmap has two channels. CNNs are well-suited for recognizing spatial patterns, so by converting each feature into a heatmap, the network can take them as input and detect changes over time. In this study, the first channel of the heatmap represents the feature values obtained from VBM analysis, and the second channel shows the rate of change of each feature over time.

C_1_: feature value (TIV-normalized, min–max scaled to [0,255] and quantized),

C_2_: rate of change (per-bin slope ΔS/Δ

t, min–max scaled to [0,255]);

1 $$c_{2}=\Delta V/T,\Delta V=\Delta S/\Delta T=(S\left(t_{2}\right)-s\left(t_{1}\right)/\left(t_{2}-t_{1}\right)$$

Where ΔS is the change in the feature, Δt is the time interval, and T = 180 months is the total study duration. Because scans in the ADNI3 database were not collected at the same times for all subjects, we chose the smallest interval (Δt = 3 months) as the step size and created a mask.

C_3_: availability mask (1 if a scan exists in the bin, else 0)

In the mask, a value of 1 means a scan exists at that time point, while 0 means no scan is available. These mask values form the third channel of the heatmap. This mask ensured the model could differentiate between missing data and zero-valued changes. For normalization, we used the following formula:

(2) $$f_{z}=\left[\frac{f-f_{\min}}{f_{\max}-f_{\min}}\right]\times 255$$

We have chosen the approach to scale all feature values to a common range [0, 1], which helps the network learn more efficiently and ensures that features with larger absolute values do not dominate the training process. Then we have applied quantization to the value stream (C_1_); the mask (C_3_) is left as binary at multiple bit depths (2, 3, 4, 8, and 16 bits), corresponding to 4, 8, 16, 256, and 65,536 intensity levels, respectively.

### 2.6 Classification and detection by deep learning algorithms

We have proposed two types of neural networks: CNN and FCN, based on their distinct capabilities in data analysis. CNNs are ideal for extracting spatial features from medical images and are well-suited to MRI heat-maps that summarize whole-brain structure, and FCNs show better performance for comprehensive data analysis and classification based on spatial details. For the phase of detection from classification, the new idea is that the features that aid in the highest accuracy in deep learning models have the potential to serve as early biomarkers for Alzheimer's disease, either signifying long-duration or fast-evolving brain atrophic changes. These informative features are deserving of further investigation. Traditional CNNs work as blind models, extracting patterns without prior knowledge. However, we introduced a new method that modifies this structure using biological features as inputs to the models. This approach has two main goals. First, it lets us measure the individual contribution of each brain feature to early AD detection by watching how the CNN's spatial filters respond. Second, by fusing the four channels, we encourage the networks to move beyond simple classification and focus on disease-specific structural change. Running the same experiment with the FCN offers a second line of evidence: when both models highlight the same feature map, confidence grows that the underlying tissue change is real.

#### 2.6.1 CNN classification

For classification, we have proposed a CNN model, based on its ability to effectively capture spatial and temporal patterns in the heatmaps, which encode both feature dynamics and data availability across time bins. We have designed a new structure of CNNs using biological features as inputs to the models. The new frame is represented in Fig. 3. This approach has two main goals. Firstly, to find the importance of each brain feature in detecting early Alzheimer's disease using CNNs to identify spatial patterns. Secondly, to improve CNNs, they not only classify AD but also detect disease-related brain changes by incorporating biological information. The model had an input layer of shape (1 row × 28-time bins × 3 channels), followed by two convolutional layers with 32 and 64 filters (3 × 3 kernel, ReLU activation, same padding), each with 2 × 2 max pooling to reduce spatial size while keeping important features. The output was flattened, passed through a dense layer with 128 units (ReLU), and finally fed into a softmax layer for five-class classification (AD, CN, MCI, EMCI, LMCI). To prevent overfitting, we used three strategies: 1) A simple model architecture with only two convolutional layers to limit complexity. 2) Stratified train-test splitting (80:20) to keep class distribution balanced. Plus, the model was trained separately for each feature for 70 epochs with a batch size of 32, using the Adam optimizer (learning rate 0.001) and sparse categorical cross-entropy loss, with accuracy as the evaluation metric.

**Figurer 3:**
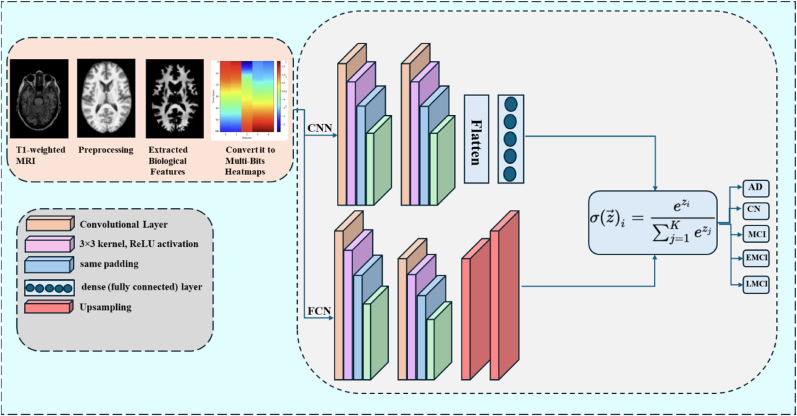
Proposed CNN-FCN model architecture for multi-class classification of AD, CN, MCI, EMCI, and LMCI. Using T1-weighted MRI images.

#### 2.6.2 FCN classification

To validate our results, we also used an FCN alongside a standard CNN. This allowed us to compare pixel-level segmentation accuracy (FCN) with image-level classification (CNN). The FCN takes heatmaps of brain features over time as input (shape: 1 × W × 4), then compresses and restores that information to predict every “pixel”. This lets the model pinpoint subtle brain changes linked to Alzheimer's disease, using both spatial patterns and biological data. After squashing the input via two convolutions + pooling layers (32 then 64 filters), a middle layer captures deep features (128 filters). Then two up-sampling steps restore the original shape, followed by convolution layers (64 then 32 filters), and finally a 1 × 1 convolution with softmax gives a probability map across five diagnostic classes (AD, CN, MCI, EMCI, LMCI), outputting (W, 4, 5). To keep the model simple and avoid overfitting, we used only two conv layers each in encoder and decoder, trained with an 80:20 stratified split, ran 50-epoch training per feature (batch size 32), with Adam optimizer (LR = 0.001), using sparse categorical cross-entropy loss and pixel-level accuracy. This setup lets us learn both individual spatial patterns and group-level differences in brain features effectively.

### 2.7 Grad-CAM visualization

In the final stage, we have used the CNN and FCN to classify diagnostic groups and to see how each feature contributes to disease progression. To do this, we applied Grad-CAM, a technique that shows which parts of the input heatmaps most influenced the model's decision. Grad-CAM works by computing gradients of the predicted class score with respect to the last convolutional layer, producing class-specific heatmaps that highlight important time points and features. This helped us understand how features like CSF_pct_TIV_calc or cortical thickness (CT_session_mm) help distinguish groups such as AD and CN, and how their changes over time relate to disease progression. For each feature, we selected a test sample heatmap and applied Grad-CAM to generate a map showing the most influential regions. Gradients from the final convolutional layer were weighted by their impact on the predicted class and averaged across spatial dimensions to create a two-dimensional heatmap. These heatmaps were normalized to [0, 1] and visualized using a jet colormap, with the x-axis showing time bins (0–84 months) and the y-axis representing the feature.

## 3 Results

The structural difference between a CN, EMCI, MCI, LMCI, and an AD subject is shown in Fig. 1 and Table 3. The image presented shows T1-weighted MRI scans in a heatmap overlay format of (VBM) results for five subject groups: The intensity differences are represented on the color bar to the right, indicating regional gray matter density or degree of atrophy, where red and yellow are higher values and blue is lower values. In Table 3, we observe a clear disease-severity gradient across groups. In the case of Gray matter (GM), related measures show larger values and faster change as impairment advances: AD exhibits GM change ranges of 452–755 mm^3^ with a faster enlargement over time, consistent with ventricular expansion tracking progression (28–46.4 % of TIV). LMCI and MCI show moderate enlargement (LMCI 452–566 mm^3^, 32–41 % TIV; MCI 573.0–667.4 mm^3^), while EMCI already demonstrates decline over time (p < 0.01; 571–755 mm^3^, 42–46 % TIV), highlighting a transitional stage with higher inter-individual variability. Cortical thickness thins most in AD (1.99–2.52 mm, 2.22 ± 0.150 mm; p < 0.001), often left-lateralized; LMCI shows a progressive decrease with temporoparietal emphasis (1.95–2.32 mm, 2.30 ± 0.249 mm), and MCI presents a moderate decrease (2.28–2.684 mm, 2.28 ± 0.404 mm). EMCI shows an early but significant thinning (p < 0.01; 2.33–2.52 mm, 2.40 ± 0.101 mm) amid variability, whereas CN remains largely stable (2.22–2.51 mm, 2.33 ± 0.177 mm; minimal longitudinal change, p < 0.05). White matter (WM) follows the same gradient: AD shows a significant reduction (p < 0.01; 461.0 ± 66.5, 29–37 % TIV), LMCI a gradual decrease (479.0 ± 76.8, 25–33 % TIV), MCI a moderate decrease (465.0 ± 96.6), EMCI variable/incomplete change (481.0 ± 65.2, 30–49 % TIV), and CN stability over time (464.0 ± 80.7, 30–36 % TIV). CSF and WMH metrics reinforce this pattern and add longitudinal context. CSF volume expands most in AD (245.5–603.8 ml), with a median +14.61 ml/year (IQR +9.57 to +16.42; 14/15 increasing; typical CSF%TIV 23.6–36.7 %). LMCI and MCI show progressive enlargement (LMCI 248.0–713.4 ml, +7.98 ml/year [4.62–15.43], 23/25 increasing; MCI 58.8–896.5 ml, +6.39 ml/year [4.21–10.93], 22/25 increasing), while EMCI already shows an early-stage increase (232.3–526.6 ml, +5.98 ml/year [4.05–7.84], 19/20 increasing). CN demonstrates age-related expansion (247.7–712.3 ml, +7.16 ml/year [4.87–8.29], 21/22 increasing), with the broadest CSF%TIV spread (24.4–46.5 %). WMH burden also rises with disease: AD shows 0.86–71.27 ml with +0.725 ml/year (IQR +0.104 to +3.204; 14/15 increasing; typical WMH%TIV 0.078–2.789 %), LMCI 0.00–16.73 ml with +0.118 ml/year (0.059–0.256; 21/25 increasing), MCI 0.00–125.40 ml with +0.189 ml/year (0.071–0.484; 23/25 increasing), EMCI 0.30–35.28 ml with +0.361 ml/year (0.122–0.724; 20/20 increasing), and CN 0.00–22.04 ml with +0.122 ml/year (0.050–0.266; 19/22 increasing). Together, these multimodal readouts depict a coherent trajectory: earliest thinning and CSF/WMH changes in EMCI, progressive divergence through LMCI and MCI, and the largest abnormalities in AD, with CN showing the expected aging patterns but markedly lower rates and magnitudes of change.

**Table 3:** Brain structural differences between groups.

Category	Group	Raw Measurement	Percentage of TIV	Trend Over Time & Significant Areas of Change
GM Volume Changes	AD	452–755 mm^3^	28 %–46.4 %	Faster enlargement over time | Ventricular expansion tracks progression
	LMCI	452–566 mm^3^	32 %–41 %	Moderate enlargement | —
	MCI	573.0–667.4 mm^3^	38 %–444 %	Moderate enlargement | —
	EMCI	571–755 mm^3^	42 %–46 %	Decrease over time (p < 0.01) | Transitional stage, higher variability
	CN	517–642 mm^3^	30 %–37 %	Minimal change | No significant atrophy; normal aging pattern
Cortical Thickness Changes	AD	1.99–2.52 mm	2.22 ± 0.150	Significant decrease (p < 0.001) | Often greater on the left
	LMCI	1.95–2.32 mm	2.30 ± 0.249	Progressive decrease | Temporoparietal emphasis
	MCI	2.28 mm, 2.684 mm	2.28 ± 0.404	Moderate decrease | —
	EMCI	2.33–2.52 mm	2.40 ± 0.101	Moderate decline (p < 0.01) | Inter-individual variability
	CN	2.22–2.51 mm	2.33 ± 0.177	Little longitudinal change (p < 0.05) | Stable cortical thickness
WM Changes	AD	461.0 ± 66.5	29 %–37 %	Significant reduction (p < 0.01) | —
	LMCI	479.0 ± 76.8	25 %–33 %	Gradual decrease | —
	MCI	465.0 ± 96.6	–	Moderate decrease | —
	EMCI	481.0 ± 65.2	30 %–49 %	Variable, incomplete change | —
	CN	464.0 ± 80.7	30 %–36 %	Stable over time | —
CSF Volume Changes	AD	245.5–603.8 ml	20.8–38.6 %	Rapid CSF expansion; median +14.61 ml/yr (IQR +9.57 to +16.42); 14/15 subjects show increases; typical CSF%TIV 23.6–36.7 %.
	LMCI	248.0–713.4 ml	17.3–85.6 %	Gradual enlargement; median +7.98 ml/yr (IQR +4.62 to +15.43); 23/25 increasing; wide spread with rare extremes (CSF%TIV 5–95 % = 20.1–34.7 %, max 85.6 %).
	MCI	58.8–896.5 ml	12.8–79.0 %	Steady rise; median +6.39 ml/yr (IQR +4.21 to +10.93); 22/25 increasing; very broad absolute range indicates heterogeneity (CSF%TIV 5–95 % = 20.3–34.8 %).
	EMCI	232.3–526.6 ml	18.9–34.1 %	Early-stage increase; median +5.98 ml/yr (IQR +4.05 to +7.84); 19/20 increasing; CSF%TIV concentrated 20.2–32.2 % (5–95 %).
	CN	247.7–712.3 ml	22.3–59.5 %	Age-related expansion; median +7.16 ml/yr (IQR +4.87 to +8.29); 21/22 increasing; broader CSF%TIV spread 24.4–46.5 % (5–95 %).
WMH Volume Changes	AD	0.86–71.27 ml	0.063–4.138 %	WMH burden climbs; median +0.725 ml/yr (IQR +0.104 to +3.204); 14/15 increasing; WMH%TIV usually 0.078–2.789 %, with high outliers.
	LMCI	0.00–16.73 ml	0.000–1.200 %	Small but progressive accumulation; median +0.118 ml/yr (IQR +0.059 to +0.256); 21/25 increasing; WMH%TIV 0.033–0.777 % (5–95 %).
	MCI	0.00–125.40 ml	0.000–8.478 %	Increase with marked heterogeneity; median +0.189 ml/yr (IQR +0.071 to +0.484); 23/25 increasing; extremes reach 125.4 ml (8.478 % TIV).
	EMCI	0.30–35.28 ml	0.022–2.356 %	Early-stage rise; median +0.361 ml/yr (IQR +0.122 to +0.724); 20/20 increasing; WMH%TIV typically 0.039–1.336 % (5–95 %).
	CN	0.00–22.04 ml	0.000–1.432 %	Low-level accumulation; median +0.122 ml/yr (IQR +0.050 to +0.266); 19/22 increasing; WMH%TIV 0.077–0.702 % (5–95 %).

### 3.1 Statistical analysis results

Descriptive statistics showed clear differences in both central tendency and dispersion across groups for all five measures (Table 4). Normality checks confirmed widespread departures from Gaussian assumptions for most variables within most groups, reinforcing the decision to adopt a nonparametric strategy (Table 5). Consistent with these diagnostics, Kruskal–Wallis tests results at Table 6 indicated significant group effects for every measure examined: CSF (χ^2^ = 54.969, df = 4, p = 3.30 × 10^−11^), GM (χ^2^ = 47.402, df = 4, p = 1.26 × 10^−9^), WM (χ^2^ = 10.923, df = 4, p = 0.0275), CT (χ^2^ = 92.791, df = 4, p < 2.2 × 10^−16^), and WMH (χ^2^ = 21.743, df = 4, p = 2.25 × 10^−4^). These omnibus results indicate that the distributions of each measure differ across the diagnostic categories.

**Table 4:** Descriptive statistics: mean (SD) for each measure by group.

Group	CSF	GM	WM	CT	WMH
AD	455.0 (79.6)	558.0 (50.1)	461.0 (66.5)	2.22 (0.150)	8.22 (12.8)
CN	443.0 (83.8)	593.0 (58.1)	464.0 (80.7)	2.33 (0.177)	3.55 (3.69)
EMCI	392.0 (63.8)	615.0 (51.2)	481.0 (65.2)	2.40 (0.101)	4.48 (6.53)
LMCI	400.0 (82.8)	575.0 (86.6)	479.0 (76.8)	2.30 (0.249)	3.13 (3.38)
MCI	397.0 (111.0)	573.0 (94.4)	465.0 (96.6)	2.28 (0.404)	4.68 (14.0)

**Table 5:** Shapiro–Wilk normality p-values by group and measure.

Group	CSF	GM	WM	CT	WMH
AD	0.175	0.738	0.179	0.505	1.73 × 10^−13^
CN	1.15 × 10^−4^	6.50 × 10^−5^	5.74 × 10^−9^	7.01 × 10^−15^	3.47 × 10^−15^
EMCI	2.22 × 10^−3^	2.10 × 10^−3^	4.94 × 10^−5^	5.68 × 10^−4^	1.41 × 10^−15^
LMCI	7.27 × 10^−4^	6.39 × 10^−13^	1.69 × 10^−8^	2.23 × 10^−21^	1.43 × 10^−17^
MCI	3.75 × 10^−7^	2.24 × 10^−17^	2.19 × 10^−14^	4.87 × 10^−21^	1.10 × 10^−24^

Values < 0.05 indicate departure from normality.

**Table 6:** Kruskal–Wallis group comparisons.

Measure	χ^2^	df	p-value
CSF	54.969	4	3.30 × 10^−11^
GM	47.402	4	1.26 × 10^−9^
WM	10.923	4	0.0275
CT	92.791	4	<0.001
WMH	21.743	4	0.000225

### 3.2 CNN and FCN results

**Fig. 4:**
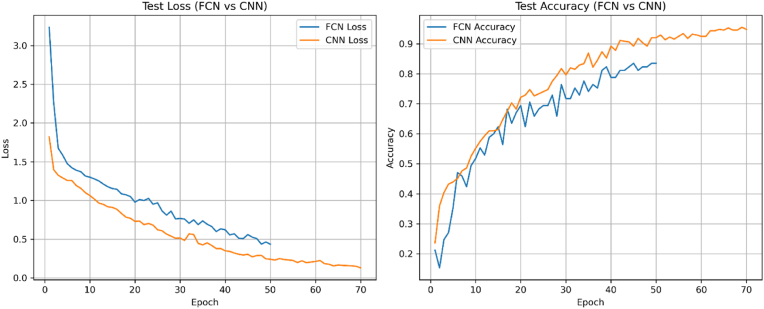
The overall framework of the proposed model: This diagram illustrates the architecture of both the CNNs and the FCNs as applied in our classification study.

We have used the CNN and FCN to classify different neurodegenerative disease groups: Alzheimer's Disease (AD), Cognitively Normal (CN), Mild Cognitive Impairment (MCI), Early MCI (EMCI), and Late MCI (LMCI), based on brain imaging data from the ADNI dataset. We have trained the model separately for five types of features: cerebrospinal fluid (CSF) percentage of total brain volume, cortical thickness, gray matter percentage, white matter percentage, and white matter hyperintensity percentage. Its performance was tested on 20 % of the data using accuracy, loss, precision, recall, and F1-score, and results were averaged across all features. The performance metrics for each feature and the overall average are presented in Fig. 4 and Table 7. The models were evaluated on a test set with a batch size of 32 and trained for 70 epochs, using a stratified train-test split to ensure balanced representation of classes. Among the features, GM_pct_TIV_calc showed the best performance, achieving 99.07 % accuracy, 99.11 % precision, 99.07 % recall, and a 99.06 % F1-score, with the lowest loss (0.0442), indicating that gray matter volume is highly discriminative for classifying neurodegenerative groups. CT_session_mm also performed very well, with 98.13 % accuracy and an F1-score of 98.13 %.

**Table 7:** Performance of CNN and FCN Models Across Metrics. (in percentage).

Feature	Accuracy (%) $\frac{TP+TN}{TP+TN+FP+D}$	Precision (%) $\frac{\text{TP}}{\mathrm{TP}+\mathrm{FP}}$	Recall (%) $\frac{\text{TP}}{\mathrm{TP}+\mathrm{FN}}$	F1 Score (%) $\frac{\mathrm{Precision}\times Recall}{\mathrm{Precision}+Recall}$
Thickness				
CNN	0.9813	0.9817	0.9813	0.9813
FCN	0.9800	0.9820	0.9800	0.9810
GM				
CNN	0.9907	0.9911	0.9907	0.9906
FCN	0.9438	0.9521	0.9307	0.9305
CSF				
CNN	0.9159	0.9177	0.9159	0.916
FCN	0.8941	0.9000	0.8941	0.8970
WMH				
CNN	0.8879	0.8952	0.8879	0.8865
FCN	0.8200	0.8250	0.8200	0.8225
WM				
CNN	0.972	0.9741	0.972	0.916
FCN	0.7800	0.7850	0.7800	0.7825

highlighting cortical thickness as a strong predictor. WM_pct_TIV_calc and CSF_pct_TIV_calc achieved solid results, with accuracies of 97.20 % and 91.59 % and F1-scores above 91 %, showing their usefulness for classification. WMH_pct_TIV had the lowest performance, with 88.79 % accuracy, 89.52 % precision, 88.79 % recall, and an F1-score of 88.65 %, and a higher loss (0.3200), suggesting that WMH is less discriminative or more variable across groups. After analyzing the features with the CNN, the FCN was used in a non-convolutional manner. Among the features, cortical thickness (CT_session_mm) showed the best performance, achieving 98.00 % accuracy, 98.20 % precision, 98.00 % recall, and a 98.10 % F1-score, with a loss of 0.0700, indicating that cortical thickness is a highly discriminative feature for classifying neurodegenerative groups. Gray matter percentage (GM_pct_TIV_calc) also performed strongly, with 94.38 % accuracy, 95.21 % precision, 93.07 % recall, and a 93.05 % F1-score, with an estimated loss of 0.0200, showing its effectiveness in distinguishing disease groups.

### 3.3 Grad-CAM results

**Fig. 5:**
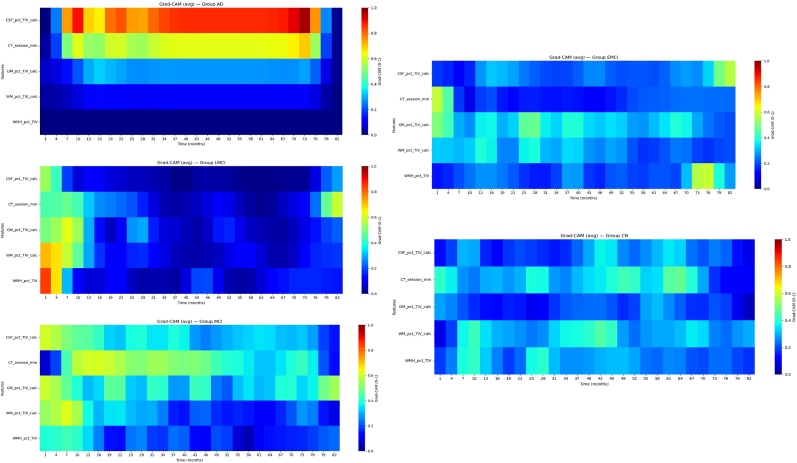
The Grad-CAM results for the CN, EMCI, MCI, LMCI and AD groups for all selected featurs

The Grad-CAM heatmap represents the average activation for five groups in the context of analyzing AD progression using CNN (Fig. 5). The vertical axis includes various features (such as CSF_pct, TIV_calc, GM_pct, WM_pct, and MRI_pct), while the horizontal axis represents time (in months), ranging from 1 to 82 months. The color scale, ranging from dark blue (value near 0.0) to red (value near 1.0), indicates the intensity of the features' influence on prediction.

## 4 Discussions

This research explored the possibility of integrating VBM with deep learning methods for the diagnosis of AD, focusing specifically on the ability to distinguish structural brain alterations that distinguish between cognitively normal (CN), Early and late mild cognitive impairment (E/LMCI), and AD. Using a carefully curated MRI dataset and methodologically sound analyses, the findings present strong evidence in favor of the use of this hybrid method. In simple terms, our CNN learned that subtle cortical thinning and gray matter loss are the tell-tale signs in early stages (e.g., in EMCI/MCI), whereas gross brain atrophy (ventricular expansion) and small vessel damage (WMH lesions) become the dominant indicators in later stages (like LMCI and AD).

### 4.1 Assessment of the dataset and VBM preprocessing

Fig. 1 depicts the preprocessing pipeline of the T1-weighted MR images, involving spatial normalization, segmentation, modulation, and smoothing. The standardized procedure follows traditional VBM protocols and maintains comparability across subjects by aligning individual brain scans into a shared space. Use of 8-mm FWHM smoothing, marked in the schematic, is best in achieving a balance between spatial resolution and signal-to-noise ratio and allows for more robust group-level inference. Of note, modulation does not destroy the volume data, which is critical in detecting atrophic patterns of AD. Our CNN learned that subtle cortical thinning and gray matter loss are the tell-tale signs in early stages (e.g., in EMCI/MCI), whereas gross brain atrophy (ventricular expansion) and small vessel damage (WMH lesions) become the dominant indicators in later stages (like LMCI and AD). By converting each person's longitudinal MRI features into time × feature “heatmaps” and applying Grad-CAM interpretability, we visualized when each feature mattered most for the model's decisions. The resulting importance maps showed that CT and GM features lit up at earlier time points for prodromal AD cases, while CSF volume (i.e., ventricle size) and WMH volume lit up at later time points for more advanced cases. This temporal shift aligns well with the known cascade of AD biomarkers: pathological amyloid and tau changes occur first (though not measured by our MRI-based model), then structural neurodegeneration in the cortex emerges, and finally widespread tissue loss and accumulated vascular injury appear as the disease progresses (Hadjichrysanthou et al., 2020; Jack et al., 2010). It is important to note that the CSF in our study refers to volumetric MRI measures of cerebrospinal fluid spaces (ventricular volume), a marker of neurodegeneration rather than the molecular CSF biomarkers (amyloid-beta or tau). Thus, the fact that MRI-CSF volume became important only at later stages in our model is biologically consistent: enlarging ventricles reflects late-stage atrophy rather than early amyloid/tau changes. Indeed, faster ventricular expansion is recognized as a sensitive marker of disease progression in AD (Nestor et al., 2008), often paralleling cognitive decline over time (Nestor et al., 2008). Our model's behavior, focusing on cortical atrophy early and ventricular/WMH changes later, echoes the clinical course of AD and lends credibility to the CNN's learned strategy.

### 4.2 Model performance

The proposed approach proved to be effective in improving the performance of deep learning models for neuroimaging analysis. The pattern discovered by our CNN meshes strongly with decades of Alzheimer's research. Early in the disease (even before dementia), one of the hallmark MRI signs is atrophy in specific cortical and limbic regions. For example, regionally specific cortical thinning (especially in temporal and parietal lobes) has been detected in very mild AD and even in asymptomatic older adults with AD pathology (Dickerson et al., 2009). Dickerson et al. reported that thinning in key AD signature cortical areas correlates with symptom severity in the earliest clinical stages, and they found subtle cortical atrophy present even in cognitively normal individuals who have amyloid in the brain (Dickerson et al., 2009). This aligns perfectly with our finding that CT and GM features were the model's focus for distinguishing EMCI/MCI from healthy aging. In essence, the CNN independently “rediscovered” that losing cortical tissue is a pivotal early marker of incipient AD. Likewise, hippocampal and medial temporal lobe atrophy are well-known to emerge around the MCI stage or earlier. Large cohort studies suggest that hippocampal volume loss can be observed as soon as amyloid starts accumulating, nearly 15–20 years before overt dementia (Hadjichrysanthou et al., 2020). It follows that our model, which did not explicitly use amyloid or tau data, relied on MRI indicators of neurodegeneration (like cortical thinning) to catch those early cases on the AD continuum. By contrast, ventricular enlargement, essentially the flip side of whole-brain atrophy, is minimal in presymptomatic stages but accelerates as the disease advances (Nestor et al., 2008). ADNI-based analyses have shown that patients with AD have significantly higher rates of ventricular expansion than MCI patients, who in turn expand faster than healthy controls (Nestor et al., 2008). Notably, MCI patients who progress to AD tend to have greater ventricle enlargement over time than those who remain stable (Nestor et al., 2008), and this enlargement correlates with worsening cognition (for instance, more rapid ventricle growth accompanies bigger declines on ADAS-Cog scores) (Nestor et al., 2008). Our Grad-CAM results mirror this trajectory: the CNN zeroed in on CSF/ventricle features when classifying late MCI and AD, effectively picking up the MRI signal of widespread brain atrophy that marks late-stage progression. The same logic applies to the WMH lesions. While WMHs are not a primary Alzheimer's pathology, they increase with age and vascular risk factors and have been consistently linked to cognitive impairment in older adults. Individuals with AD or MCI often show elevated WMH burden (especially in posterior regions), and higher WMH volumes predict greater risk of converting from MCI to dementia as well as faster cognitive decline (Brickman, 2013). Brickman (2013) emphasized that WMH might serve as an independent contributor to cognitive impairment or synergize with “primary” AD pathological changes to worsen outcomes (Brickman, 2013). This provides a biological rationale for what our model learned: in early stages, WMH volume is low and not very discriminatory, but in later stages, a high WMH burden is a red flag. Indeed, our CNN largely ignored WMH features for young AD patients or EMCI cases (who likely had few lesions), but for older LMCI/AD patients with substantial small vessel disease, WMH became an important piece of the classification puzzle. In summary, according to our model, CNN's temporal feature importance aligns with the known sequence of AD-related changes: from initial subtle neurodegeneration in cortex and hippocampus, to later global brain loss and vascular comorbidity. This concordance with the literature not only validates our model's.

findings but also reinforces existing theories of AD's progression(Jack et al., 2010; Dickerson et al., 2009). Below, we summarize the key findings across four feature sets, cortical thickness, GM, CSF, WM, and WMH, followed by class-specific performance for Alzheimer's disease (AD), CN, EMCI, and LMCI.

#### 4.2.1 Integrating biological features and model interpretability

Table 8 summarizes the model results for CNN and FCN across different features. The comparison is based on confusion matrices, using classification accuracy and misclassification trends. Overall, CNN outperforms FCN, particularly in distinguishing AD and CN, though both models are effective, with FCN showing higher misclassification rates across MCI stages. Based on Table 8, the performance of CNN and FCN models across Cortical Thickness, GM, CSF, and WM features is analyzed. CNN demonstrates near-perfect classification for AD, MCI, and LMCI in Cortical Thickness with minimal misclassifications, while FCN shows strong performance but with slightly higher errors, especially between EMCI and LMCI. For GM, CNN achieves perfect AD and CN classification with rare MCI/LMCI errors, whereas FCN struggles more with these latter classes, reducing its accuracy. In CSF, both models perform well, but CNN has fewer CN/LMCI confusions, while FCN exhibits slightly more. For WM, CNN shows higher AD/CN misclassifications yet maintains high recall for AD and LMCI, while FCN experiences significant AD/CN and LMCI/MCI confusion, leading to lower recall. Overall, CNN outperforms FCN, particularly in distinguishing AD and CN, though both models are effective, with FCN showing higher misclassification rates across MCI stages.

**Table 8:** Summarizing the model evaluation results for CNN and FCN across different features.

Feature	Key Misclassification Notes
Cortical Thickness	
CNN	Near-zero errors across AD, MCI, LMCI
FCN	Higher Errors between EMCI and LMCI
Gray Matter	
CNN	Perfect AD/CN, rare MCI
FCN	More EMCI/LMCI errors
Cerebrospinal Fluid	
CNN	Rare CN/LMCI confusion
FCN	Slightly more CN/LMCI errors
White Matter	
CNN	Higher AD/CN errors
FCN	Significant AD/CN, LMCI/MCI confusion
**WMH**	
CNN	Accurate AD/CN, minor errors
FCN	More MCI/LMCI confusion

#### 4.2.2 CNN decisions via Grad-CAM

A key advantage of our framework is that it marries high accuracy with human-interpretable insight. We have used the Grad-CAM to identify what aspects of the time-series heatmap drove the CNN's decisions. Normally, Grad-CAM is applied to image classification problems to highlight which image regions influenced the prediction. In our case, the “image” is a 2D matrix of time (x-axis) by feature (y-axis), so Grad-CAM highlights when and which features are most important for the model's output. Generally, for each subject, Grad-CAM produces a coarse map indicating the contribution of each time-point in each feature channel to the final classification (Selvaraju et al., 2019). By averaging these maps over many individuals in the same diagnostic group, we obtained smooth, denoised profiles that are easy to interpret. In practical terms, these temporal heatmaps told us that, for example, the model identified this person as EMCI. Their gray matter volume dropped notably in the first year, or the model flags this patient as likely AD because their ventricles expanded rapidly in the last two visits. Such statements, derived from the Grad-CAM visualization, are remarkably intuitive; they resonate with clinical expectations about what changes signal progression. This level of interpretability is seldom available in deep learning models, which are often criticized as “black boxes”. Here, however, we can literally point to the evidence the model used, in a timeline format. It builds confidence that CNN is making decisions for the right reasons (i.e., known disease markers) rather than spurious noise. We also took steps to ensure these Grad-CAM insights were trustworthy. We followed the recommended sanity check papers. neurips.cc, such as confirming that if we randomly re-initialize the model's weights (breaking the learned patterns), the Grad-CAM heatmaps become meaningless. This assures us that the highlighted patterns truly stem from the trained model's learned features and are not an artifact of the architecture or data scaling papers.neurips.cc. In summary, the Grad-CAM analysis turned our CNN from a complex classifier into a sort of digital expert that can explain its reasoning. It highlighted temporal areas of focus that align well with medical knowledge (early cortical changes, late ventricle/WMH changes), thereby making the model's decisions more transparent and acceptable to clinicians. We believe this approach (treating longitudinal data as an interpretable image) can be broadly useful for understanding what a deep model “sees” in patient trajectories. According to Fig. 5, in the early time period for CSF (months 1–10), yellow and green areas are visible, indicating a moderate to high influence (0.4–0.6) of these features. This may suggest the initial role of CSF in the early stages of LMCI. Furthermore, in the mid-time period of GM (around months 20–40), areas of moderate intensity (light blue) are observed, indicating that the percentage of GM and total intracranial volume still play a role, though their influence has decreased. The prominent red area for the WM_pct feature during the early time period (months 1–10), with an influence nearing 1.0, highlights the critical role of the percentage of white matter (WM) in predicting LMCI, likely reflecting substantial early structural changes or degeneration in the white matter that are detectable by the model. In contrast, the red area for the next feature in the initial months (1–5) suggests that MRI data strongly contributes to the initial diagnosis of LMCI, possibly due to its ability to capture early imaging biomarkers; however, the fading influence over time indicates that as the disease progresses, these MRI-based features may become less distinctive or are overshadowed by other factors, reflecting a shift in the disease's manifestation or the model's focus on different predictors. So, features such as white matter and WMH play a prominent role in the early stages of LMCI, potentially indicating the importance of early structural changes in white matter. Over time, the influence of these features decreases, which could suggest stabilization or a more complex disease progression. In contrast to LMCI, in the early stages (months 1–10), CT_session_mm and GM_pct display yellow-green areas (0.4–0.6), suggesting a moderate to high initial influence of session duration and gray matter percentage, possibly reflecting early detectable changes. WM_pct shows light blue areas (around 0.2 to 0.4) with occasional yellow patches, indicating a lower but variable influence of white matter, which may point to less pronounced early changes compared to LMCI. MRI_pct exhibits a yellow area around months 60–70 (0.6–0.8), suggesting a late resurgence of WMH influence, potentially due to evolving imaging biomarkers. Over time, the dominance shifts to dark blue (near 0.0) across most features, indicating a general decline in their predictive power, which could reflect stabilization or reduced detectability of changes as EMCI progresses, contrasting with LMCI's stronger early white matter influence. For a comprehensive comparison, integrating EMCI and LMCI data could clarify these trends further. The results of Grad-CAM for CNN and FCN were similar. However, since CNN achieved higher accuracy, we focused on explaining its results.

### 4.3 Comparison with existing methods and future directions

Table 9 brings together a bunch of recent deep learning methods for Alzheimer's disease. While a lot of these techniques claim high classification accuracy, they usually rely on advanced imaging protocols or heavy-duty preprocessing, and they're pretty much limited to classification alone. In contrast, our method not only maintains strong accuracy but also enables earlier detection, all through a simpler and more interpretable framework that leverages domain-specific biomarkers to identify AD-related changes sooner.

**Table 9:** Summarizing the recent deep learning methods for Alzheimer's disease.

Reference	Input Data	Technique	Limitation	Achievements
Zhou et al. (2021)	T1-weighted MRI (ADNI: 151, AIBL: 107, NACC: 565)	GAN + FCN for AD classification	Requires paired 1.5 T–3 T scans	Improved image quality, better AD classification accuracy
Bae et al. (2020)	T1-weighted MRI (ADNI, SNUBH datasets)	CNN-based AD classification using coronal slices	Performance varies by ethnicity and education level, computationally expensive.	High accuracy (AUC 0.91–0.94), rapid diagnosis (23–24s per patient), cross-population validation
Huang et al.,	Multi-modality (T1-MRI + FDG-PET) from ADNI (731 CN, 647 AD, 441 sMCI, 326 MCI)	3D CNN for AD classification without segmentation	Requires multi-modality scans, high GPU memory usage	State-of-the-art accuracy (90.1 % CN vs. AD), robust classification across modalities
Tran et al., 2023	3D brain MRI (AD-86, AD-126)	CNN + GMM segmentation + XGBoost + SVM	Reduced accuracy for anatomically challenging datasets	Accuracy: 88 % (AD-86); Dice coefficient: 0.96
Wen et al., 2020	OASIS MRI Dataset (416 images)	Deep CNN (binary AD vs. CN)	Small validation dataset	Accuracy: 93.18 %
Cui and Liu, 2018	T1-weighted MRI (ADNI)	Hippocampus segmentation + 3D DenseNet (AD vs. CN, MCI)	Moderate accuracy for MCI vs. NC (76.2 %)	Accuracy for AD vs. NC: 88.9 %; AUC: 92.5 %
Our Proposed Method 2025	T1-weighted MRI (ADNI)	Voxel-based morphometry + CNN + FCN with biologically extracted features transformed into multi-bit images (3-bit, 4-bit, 8-bit)	Dependence on the quality of feature extraction	CNN achieved 98 % accuracy, FCN achieved 84 % accuracy.

### 4.4 Limitations and future directions

We acknowledge several limitations in our study, which also point toward avenues for future work. First, in our experimental setup, we trained separate CNN models for each feature type (CT, GM, CSF volume, WMH) rather than a single integrated model. We chose this approach to simplify interpretation, which allowed us to clearly see the contribution of each biomarker over time. However, in reality, the progression of AD is driven by multiple factors interacting together. An important next step will be to develop a joint multi-modal model that ingests all feature channels at once, so that the CNN can learn combined patterns (for example, how concurrent cortical thinning *and* WMH accumulation might jointly indicate a certain trajectory). Such a model could potentially improve overall accuracy and reveal interactions between biomarkers, though interpreting the Grad-CAM from a multi-channel input will be more complex (perhaps requiring techniques to disentangle which channel drove a given activation). Second, our handling of time in the heatmaps (using fixed 3-month bins across individuals) is an approximation. Real patients have irregular visit intervals, and disease progression is not perfectly linear or synchronized. We mitigated this by including the mask channel (so the model knows which time points are missing) and by using short bin widths to capture fine changes, but some temporal nuance is inevitably lost. Future models might employ recurrent neural networks or transformers that can directly handle irregular time intervals, or use continuous-time modeling to better align patients who have different follow-up schedules. Third, our definition of “CSF” was limited to MRI-based volume measures of the ventricles and sulcal CSF spaces, essentially a surrogate of brain atrophy. We did not include actual CSF biomarkers of amyloid or tau, nor amyloid PET or tau PET imaging, which are critical components of the modern AT(N) framework (Jack et al., 2010). Therefore, while our results fit the known ordering (amyloid→tau→atrophy) in a qualitative sense, we can't directly comment on the earliest pathological changes that happen before structural MRI changes. It would be fascinating to extend our heatmap approach to include such biomarkers (e.g., as additional channels or parallel heatmaps), to see if a deep model could learn an even more comprehensive timeline, perhaps identifying those subtle molecular changes that herald future atrophy. Another limitation is that our cohort was drawn from ADNI, which, while extensively studied, may not represent the general population. Participants in ADNI are volunteers with higher education and frequent monitoring, and our model might perform differently in a more diverse, “real-world” clinical sample. Rigidly binning data and quantizing values might also pose issues when applying to other datasets with different assessment schedules or measurement scales. Thus, external validation on independent cohorts (and possibly recalibrating the time-binning strategy) will be crucial to ensure generalizability. We also note that by collapsing each MRI modality to a single summary value per time point (e.g., total gray matter volume, total WMH volume), we lose regional information. An advanced extension could combine our approach with regional or voxel-wise measures; for instance, one could imagine a hybrid model where global trends are captured by our heatmap method, while local patterns are captured by a parallel CNN analyzing the MRI scans or regional maps directly. This could marry the strengths of global longitudinal profiling with the rich spatial detail of neuroimaging data.

Despite these limitations, our work has some clear implications for research and clinical practice. The finding that cortical atrophy markers (CT and GM) are most informative within the first 1–2 years of follow-up suggests that those measures are critical for early detection. Clinicians and trials focusing on prodromal AD might prioritize sensitive measures of cortical thickness or hippocampal volume during that window to identify individuals at high risk of progression. Conversely, the prominence of ventricular volume and WMH in later stages suggests that these markers are more useful for tracking disease progression and possibly treatment effects in patients who are already in the mild-to-moderate dementia range. For example, an increase in ventricle size over a year might be a quick MRI indicator that a patient's neurodegeneration is accelerating, or a high WMH burden might signal that vascular health needs management in conjunction with AD therapy. Our model essentially learned a data-driven version of the AD timeline, reinforcing the idea that different biomarkers dominate at different disease stages. This adds weight to the AT(N) conceptual framework proposed in Alzheimer's research (Jack et al., 2010). In the AT(N) model, amyloid (A) and tau (T) biomarkers become abnormal first, followed by neurodegeneration (N) markers like MRI atrophy. Our results support this: even without A/T data, the MRI-based neurodegenerative changes (N) were key early flags in our EMCI/MCI classification, consistent with the notion that by the time someone has mild symptoms, amyloid and tau have been brewing and now structural loss is detectable (Jack et al., 2010). Then, as the condition advances, the widespread atrophy (ventricular CSF volume), an MRI “N” marker, and additional factors like WMH contribute more to distinguishing the later stages. This nuanced understanding could help in designing stage-appropriate interventions or monitoring plans. It also underscores the value of longitudinal data: a single MRI snapshot might miss these temporal patterns, whereas serial scans allowed our model to not only improve accuracy but also tell a story of how the disease unfolds.

Future directions will focus on enhancing both the model and its clinical utility. One promising avenue is to incorporate multiple data types into the deep learning framework; for example, combining MRI with cognitive test trajectories, genetic risk factors, or fluid biomarkers. In principle, our heatmap representation could be extended so that, say, cognitive scores over time form another channel, or APOE genotype is encoded as an additional “feature” line (constant over time). A model that fuses multi-modal longitudinal data might capture AD's multifaceted nature more completely. We are also interested in moving from diagnostic classification to prognosis. Instead of training the CNN to classify current diagnoses, we could train it to predict future outcomes; for instance, will an MCI patient convert to AD in the next 2 years, or what will a patient's cognitive score be in the future? The fact that our current model's attention shifted to ventricles and WMH in later stages is encouraging, since those are known prognostic markers (e.g., ventricle expansion rate predicting conversion (Nestor et al., 2008), and WMH burden predicting faster decline (Brickman, 2013)). We suspect that by training on conversion or decline endpoints, the model might further emphasize those features, and Grad-CAM would then tell us the *temporal* signals most predictive of impending deterioration. Finally, we aim to validate our findings across different cohorts and scanning protocols. If a model trained on ADNI can generalize to other studies (or if we can fine-tune it with a small new sample), that would demonstrate robustness and pave the way for real-world application. We also plan to refine the interpretability: while our current Grad-CAM gives a high-level temporal importance, pairing it with spatial maps could be insightful. For example, if cortical thinning is important at 12 months, is it a particular region (like the entorhinal cortex) driving it? Integrating regional analyses or using layer-wise relevance propagation on the input features might help answer such questions.

## 5 Conclusion

This paper presents a strong framework for machine-based Alzheimer's disease classification and early detection through a combination of voxel-based morphometry and deep learning. Model prediction alignment, VBM group contrasts, and saliency maps lend strength to the biological plausibility of the approach. Even though MCI classification is still not a simple task, the results indicate the potential of data-driven models for this early diagnosis. The innovative approach of transforming extracted features into images was systematically evaluated across two distinct models: CNN and FCN. Thus, this makes this method very useful in complex classification tasks for pattern recognition, as it is also makes the method rather helpful for nuanced pattern recognition. Testing across these models allows us to gain full insight into performance and learn what each architecture excels in. The CNN was very good at learning complex patterns from the image data. The FCN offered significant transparency through clear decision pathways, and the FCN succeeded as a robust feature synthesis as a complement. This multi-model evaluation demonstrates that the innovative feature of image transformation is well-suited for improving classification outcomes. Our results confirm that this approach scales well across various neural network architectures, yielding both improved performance and a deeper understanding of the data structure.

## CRediT authorship contribution statement

**Mohammad Rezaei:** Writing – original draft, Methodology, Formal analysis, Data curation. **Shaghayegh Mohammadikhaveh:** Writing – review & editing, Writing – original draft, Methodology, Formal analysis. **Hadis Faraji:** Writing – original draft, Software, Methodology, Formal analysis. **Ramin Ardalani:** Writing – original draft. **Mina Rezaei:** Formal analysis. **Alireza Shirazinodeh:** Writing – review & editing, Supervision, Project administration, Methodology, Formal analysis, Conceptualization.

## Declaration of competing interest

The authors declare that they have no known competing financial interests or personal relationships that could have appeared to influence the work reported in this paper.

## Data Availability

Data will be made available on request.

## Footnotes

1 Data used in preparation of this article were obtained from the Alzheimer's Disease Neuroimaging Initiative (ADNI) database (adni.loni.usc.edu). As such, the investigators within the ADNI contributed to the design and implementation of ADNI and/or provided data but did not participate in analysis or writing of this report. A complete listing of ADNI investigators can be found at: http://adni.loni.usc.edu/wp-content/uploads/how_to_apply/ADNI_Acknowledgement_List.pdf.
